# Simultaneous Expression of Displayed and Secreted Antibodies for Antibody Screen

**DOI:** 10.1371/journal.pone.0080005

**Published:** 2013-11-11

**Authors:** Yuanping Zhou, Junjie Wang, Ivan Zhou, Haibo Lou, Chang-Zheng Li, Zhen-Rui Chen, Zhe-Huan Zhang, Shuwen Liu, Shuguang Wu, Wanlong Tan, Shibo Jiang, Chen Zhou

**Affiliations:** 1 Nanfang Hospital, Southern Medical University, Guangzhou, China; 2 School of Pharmaceutical Science, Southern Medical University, Guangzhou, China; 3 School of Traditional Chinese Medicine, Southern Medical University, Guangzhou, China; 4 Key Laboratory of Medical Molecular Virology of Ministries of Education and Health, Shanghai Medical College and Institute of Medical Microbiology, Fudan University, Shanghai, China; 5 Lindsley F. Kimball Research Institute, New York, New York, United States of America; 6 Dgen Biotech Limited, Hong Kong, China; Lindsley F. Kimball Research Institute, United States of America

## Abstract

The display of full-length antibody on the cell surface was achieved by fusing a transmembrane domain of the platelet-derived growth factor receptor (PDGFR) to the C-terminus of the heavy chain constant region. We also incorporated a furin cleavage site between the constant region and PDGFR transmembrane domain to obtain secreted antibodies. As a result, antibodies can be expressed simultaneously on the cell surface in a membrane-anchored version for screening and selecting through fluorescence-activated cell sorting (FACS) analysis, as well as in conditioned medium in a secreted version for function analysis.

## Introduction

By fusion with an anchor or a transmembrane domain (TM), a secreted protein, such as recombinant single-chain variable fragment (scFv) of antibodies, or even full-length antibodies, can be displayed on the surface of phage (phage display) [1]–[4], bacteria (bacteria display) [5] or yeast (yeast display) [6]–[9]. Phage display is currently the most developed display method for mAb screening, and numerous antibodies against a variety of antigens have been developed for different applications, but many limitations still accompany its use. In most cases, the isolated scFvs by phage display need to be converted into full-length antibodies for further development, and many of the converted immunoglobulin G (IgG) molecules lose binding ability when expressed from mammalian cells [2]–[3]. The use of different codons and lack of post-translational modification in bacteria result in a bias against all mammalian proteins, including antibodies. Therefore, further optimization in expression, affinity, and function is usually necessary for scFv, while, at the same time, the conversion and optimization of antibodies is a very time-consuming and labor-intensive process.

To address such drawbacks presented by phage display, scientists have tried very hard over the past ten years to develop mammalian display technology. By fusing a TM at the 3′-end of the heavy chain or scFv, the full-length antibody [10]–[11] or scFv [12] can be displayed on mammalian cell surfaces. Using the Flp-In™ system, each host cell expresses only one specific antibody, facilitating antibody screening and selecting [13]. Dual expression vectors containing both heavy and light chain genes can be constructed in single four-way ligation [14]. Several full-length fully human antibody display libraries with a combinational diversity of 10^9^ have been constructed [15]–[17], and antigen-specific antibodies have been identified from one of the constructed libraries [18].

Although membrane-bound antibodies are useful for fluorescence-activated cell sorting (FACS) analysis and antibody selection, secreted antibodies are also necessary for a variety of analytic experiments. Thus, it would be ideal if the antibody could be expressed in both an anchored form and a soluble form simultaneously. Furin, a cellular protease which recognizes the consensus amino acid sequence RXRR, cuts proteins that contain this sequence after the fourth R as they reach the trans-Golgi network (TGN) [19]. Previous reports have shown successful expression of virus membrane proteins in a soluble form through the insertion of a furin cleavage sequence (FCS) into the gene [20]. However, furin cleavage in cells is not a very effective process. As a result, some of the target proteins will not be cleaved and remain membrane-bound.

We have previously constructed the dual expression vector pDGB4, which contains both heavy chain and light chain expression cassette [14]. After transfection of this vector into mammalian cells, full-length antibody can be displayed on the cell surface because of the presence of a trans-membrane (TM) domain in frame at the 3′-end of heavy chain. Here we report the insertion of a sequence coding for a peptide RIRR between the heavy chain and TM into the dual expression vector pDGB4. The RIRR sequence can be recognized and cleaved by furin, a naturally inherent protease. Because of its presence, a portion of the antibodies expressed from the vector are displayed on the cell surface for screening and selection, while the cleaved portion of antibodies, which can be used for other analyses, are expressed in a soluble form in condition medium.

## Materials and Methods

### Reagents and cell lines

Restriction enzymes and T4 DNA ligase were purchased from Fermentas (Hanover, MD). Antibody reagents were purchased from BD Pharmingen (San Diego, CA). Primers were synthesized by Invitrogen (San Diego, USA). Ready-to-use Taq DNA polymerase (2 x Master Mix) was purchased from Promega (San Luis Obispo, CA). DH5α competent cells were purchased from Takara (Otsu, Shiga, Japan). The Flp-In™ system, including vector pcDNA5/FRT, vector pOG44, Flp-In Chinese hamster ovary (FCHO) cell line, and related cell maintenance media, were purchased from Invitrogen (Carlsbad, CA). 293-T cells (ATCC) were maintained in DMEM supplemented with 10% FBS.

### Construction of pDGB4-noTM and FCS-containing vector pDGB4-FCS

Both vectors were constructed by standard molecular techniques. Briefly, the vector pDGB4–noTM was constructed by deletion of the TM domain in pDGB4. To construct the vector pDGB4-FCS, forward primer (5′-GGTAAAGGATCCCGGATCAGGCGCAATGCTGTGGGC CAGGACACG-3′) and reverse primer (5′-TGGCAACTAGAAGGCACAGTCGAGGC-3′) were synthesized by Invitrogen (San Diego, USA) and used to introduce a nucleotide sequence of 12 base pairs (5′-***CGGATCAGGCGC***-3′) coding for RIRR into the vector pDGB4 in frame right before TM by PCR assembling. After PCR amplification using pDGB4 as template, the fragment was digested with BamHI and XhoI. The fragment “BamHI-RIRR-TM—XhoI” was inserted into the vector pDGB4 to replace the TM region. Following ligation and transformation, four colonies were analyzed by enzyme digestion, and 3 out of 4 contained right-sized fragments. Sequence analysis confirmed that clone numbers 2 and 3 had the FCS sequence in frame.

### Miniprep, maxiprep, gel extraction, and PCR clean-up

All were performed using the kits purchased from Axygen (Union City, CA), according to the manufacturer's directions.

### DNA digestion and fragment purification

Vector DNA was isolated from overnight *E. coli* bacterial cultures. After digestion with proper restriction enzymes, DNA fragments were separated through electrophoresis in 1% agarose-TBE gel. The target fragments were then isolated by gel extraction kit.

### PCR reaction

PCR was carried out in a total volume of 50 µl containing 200 nanomoles of forward primer and reverse primer, 50 ng of template, and 25 µl of 2x Master Mix. Amplification conditions were as follows: 94°C for 5 min to denature the template, then 30 cycles of 30 sec at 94°C, 30 sec at 55°C, and extension at 72°C for 1 minute per 1 kb length of DNA to be amplified, ending with 7 minutes of extension at 72°C. The PCR products were electrophoresed in 1% agarose-TBE gel and purified. The purified fragments were then digested according to experimental needs, purified by PCR clean-up kit, and used for ligation.

### Vector ligation and transformation

About 50 to 100 ng of total vector and insert fragments were mixed at a molecular ratio of 1∶1 (unless otherwise stated) in a total volume of 10 µl with 1 unit of T4 DNA ligase. After ligation for at least two hours at room temperature, 1 µl of ligation mixture was used in transformation with 50 µl of DH5α competent cells following the manufacturer's directions. The proper amount of bacteria was plated on LB-ampicillin plate and cultured at 37°C overnight. The colony numbers were counted and the transformation efficiency calculated.

### Transfection

Transfection was performed with either 293-T cells or FCHO cells according to experimental needs. Typically, transfection was performed in a 12-well plate, unless otherwise stated. The day before transfection, 4×10^5^ cells were seeded in each well for transfection the next morning. Two µg of DNA and 5 µg of transfection reagent (Dgen Biotech Ltd.) were separately diluted in 100 µl of DMEM each and then mixed. The mixture was incubated at room temperature for 30 minutes and then directly added into each well without changing the culture medium. After six hrs of incubation at 37°C, the medium was changed with fresh culture medium. The antibody expression was then analyzed by FACS 48 to 72 hrs post-transfection.

### FACS analysis of antibody expression on the cell surface

Cells were dissociated by cell dissociating buffer (Invitrogen), followed by one wash with staining buffer (2% FBS in PBS). Next, 5×10^5^ cells were stained by proper fluorescence-labeled anti-human IgG and/or anti-human kappa chain antibodies in 50 µl of total volume at 4°C. Finally, cells were washed and resuspended in 400 µl of staining buffer, and antibody expression was then analyzed by FACS.

### Establishment of a stably transfected cell pool

The vector containing both heavy chain and light chain was mixed with vector pOG44 in a ratio of 1∶9 and transfected into FCHO. Twenty-four hours post-transfection, the cells were split 1∶10, followed by incubation for an additional 24 hrs. The cells were selected under 500 µg/ml hygromycin B until cell clones formed. The expression of antibodies on the cell surface was analyzed by FACS, and the expression of antibodies in a secreted form was analyzed by Western blot.

### Western blot

Stably transfected FCHO cells (5×10^5^) were seeded in a 12-well plate. The media were changed and replaced by 500 µl of DMEM without FBS 24 hours post-cell seeding. The DMEM-condition media were collected 24 hrs post-media change for Western blotting analysis, and cells were dissociated for FACS analysis. The condition media were concentrated 10-fold using Microcon YM-10 (Millipore, Billerica, MA, USA). Twenty-five µl of concentrated condition media were mixed with 8 µl of 4x sample buffer and boiled for 3 min. Ten µl of denatured samples were loaded on 10% sodium dodecyl sulfate polyacrylamide gel for electrophoresis (SDS-PAGE) to separate the proteins in the media. After running in 1x SDS glycine buffer for 1 hr, the proteins in the gel were transferred to a PVDF (Polyvinylidene Fluoride) membrane. The membrane was rinsed twice with staining buffer (PBS + 0.05% Tween-20), blocked by 5% nonfat milk for 30 minutes, and then stained by HRP-conjugated goat anti-human IgG (heavy and light chain) antibodies (ZSGB-BIO, Beijing, China). After staining for 1 hour, the membrane was washed twice by staining buffer, and the target antibodies were detected by ECL kit (Cell Signaling, USA) as described by its manual.

## Results

### Insertion and sequence confirmation of furin cleavage site (FCS) in the vector

**Figure 1:**
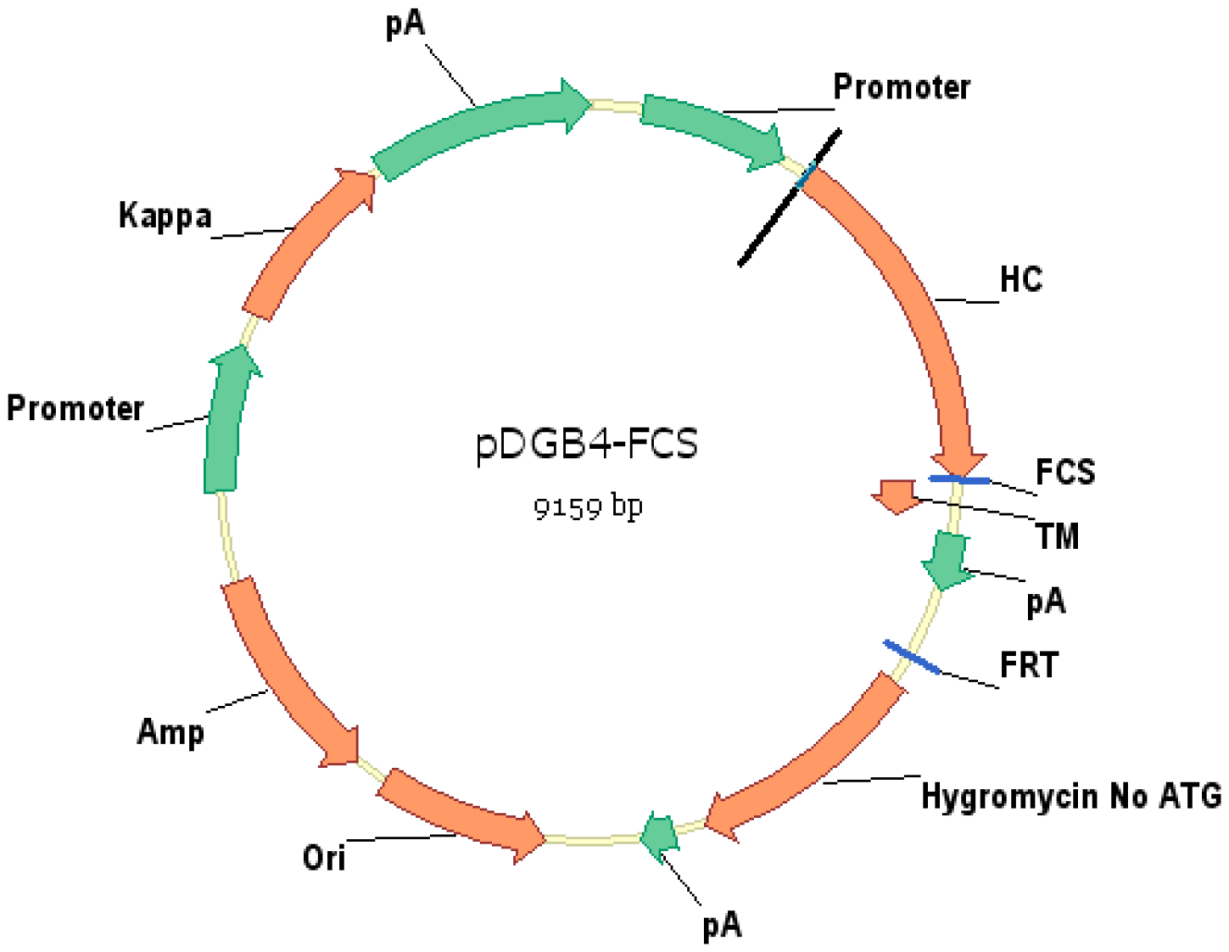
Schematic illustration of vector pDGB4-FCS. The vector contains both heavy chain and light chain expression cassettes. After integration of the vector into the genome of host cell, the full-length antibody can be expressed as both displayed and secreted versions simultaneously on cell surface as well as in condition medium.

**Figure 2:**
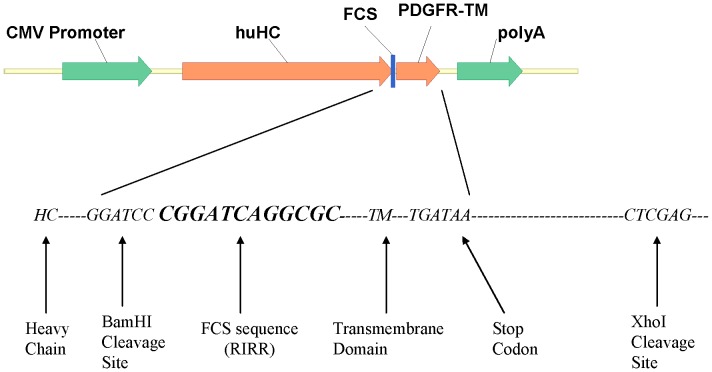
FCS diagram. The nucleotide sequence coding for Furin cleavage site RIRR is inserted between heavy chain and trans-membrane domain in frame by PCR assembling.

Vector pDGB4 has been shown previously [14] to express membrane anchored antibodies. Vector pDGB4–noTM was derived from pDGB4 by deletion of the TM domain to express soluble antibodies. Vector pDGB4-FCS was constructed to test whether insertion of FCS into pDGB4 between the heavy chain constant domain (Ch) and platelet-derived growth factor receptor (PDGFR) transmembrane domain (TM) would allow furin to cleave and remove the TM domain, thereby releasing the membrane-displayed antibodies into culture medium (Fig. 1). For this purpose, a nucleotide sequence of 12 base pairs (5′-***CGGATCAGGCGC***-3′) coding for RIRR was fused in frame right before TM as illustrated in Figure 2. Sequence analysis confirmed the presence of the FCS sequence in frame.

### Analysis of antibody expression through FCS-containing vector

To compare the antibody expression on the cell surface with and without FCS, we used the Flp-In system, including the Flp-In Chinese hamster ovary (FCHO) cell line, vector pcDNA5/FRT, vector pOG44, and related cell maintenance media, because of its ability to integrate a gene of interest into the host cell at a specific genomic location [21]. In the FCHO cells, a flippase recombination target (FRT) site has been introduced into the cell's genome, into which an expression vector containing an FRT site can be integrated via Flp recombinase-mediated DNA recombination at the single FRT site in genome. In that way, the expression of the gene of interest contained in a vector will only be dependent on that vector's characteristics, rather than other underlying variables such as copy numbers, integrated vector or integration location.

**Figure 3:**
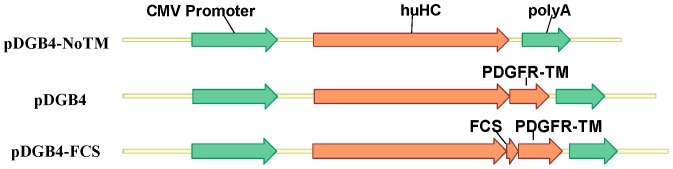
Schematic comparison of expression vectors. pDGB4 contains a trans-membrane domain at the 3′-end of heavy chain in frame and the antibody expressed is displayed on cell surface. pDGB4-noTM is derived from pDGB4 by deletion of the trans-membrane domain, and the antibody expressed is in soluble version. pDGB4-FCS can simultaneously express both displayed and secreted antibody molecules because of the presence of Furin cleavage site (FCS) between heavy chain and trans-membrane domain.

Plasmid DNAs of vectors pDGB4-FCS, pDGB4 and pDGB4-noTM were stably transfected into FCHO cells by co-transfection with pOG44 which contains the Flp recombinase gene. As shown in Figure 3, pDGB4-FCS contains both TM and FCS regions. Vector pDGB4 contains TM, but no FCS sequence, and all of the antibodies are predicted to be membrane-anchored. Vector pDGB4-noTM contains neither TM nor FCS at the end of HC, and all of the antibodies expressed should be secreted into the culture medium. All three vectors contain the same vector backbone, antibody gene and FRT site. Therefore, the amount and ratio of antibodies on the cell surface and in conditioned medium will only be dependent on the function of FCS and TM.

**Figure 4:**
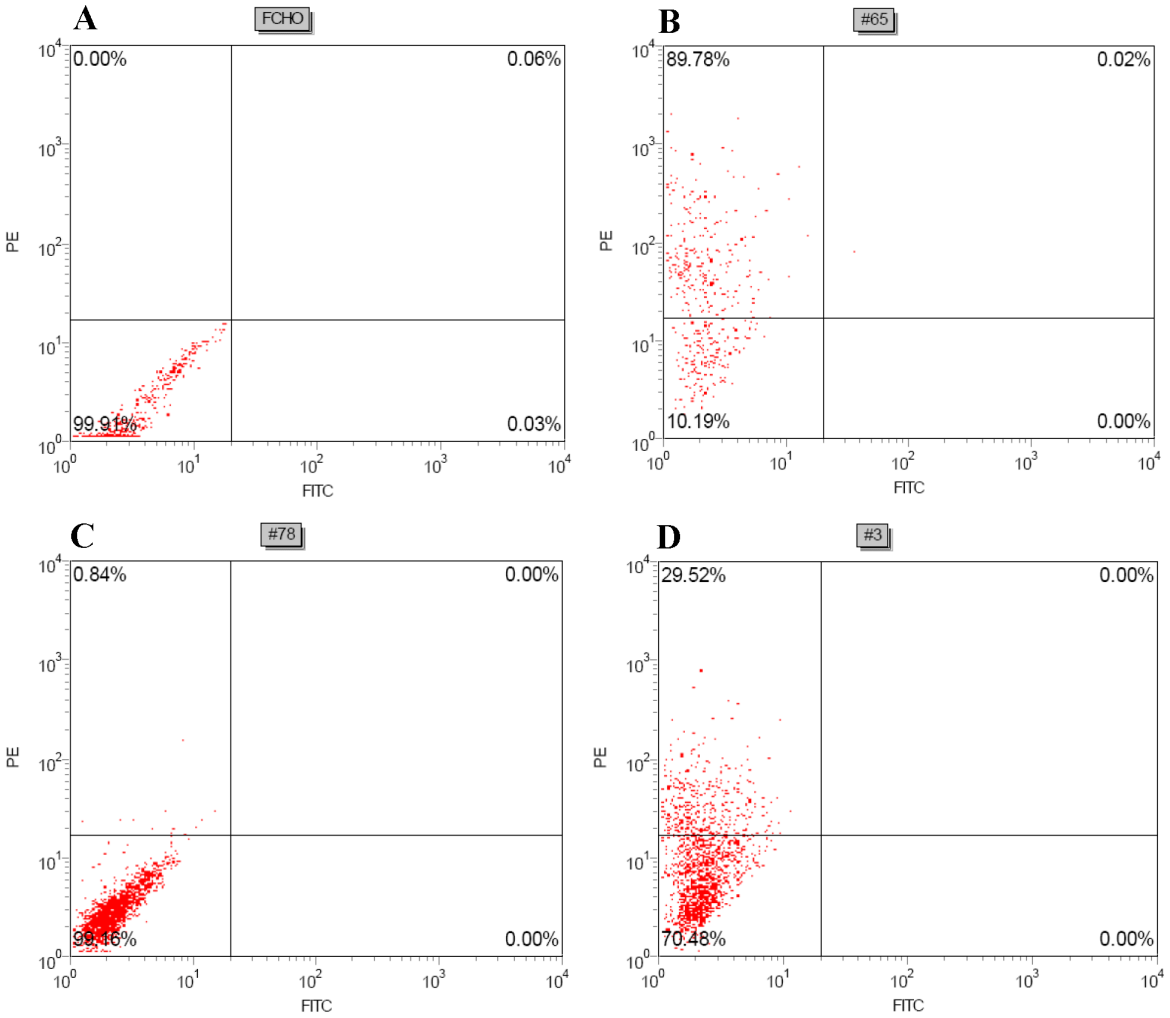
Antibody expression through FCS-containing vector. Flip-In CHO cells (FCHO) stably transfected by plasmid DNA were stained by PE-conjugated mouse-anti-human kappa chain antibodies (PE-Kappa) and then analyzed by FACS. (**A**) FCHO, no staining; (**B**) pDGB4, PE-Kappa staining; (**C**) pDGB4-noTM, PE-Kappa staining; and (**D**) pDGB4-FCS, PE-Kappa staining.

Three selected cell pools from the above vectors were stained by PE-conjugated mouse anti-human kappa chain antibody and analyzed by FACS. The results are shown in Figure 4. As expected, 90% of cells from pDGB4 were detected to express antibodies on the cell surface (Figure 4-B), while no antibody expression was detected on the cell surface of pDGB4-noTM (Figure 4-C). In contrast, 30% of cells from pDGB4-FCS have antibodies expressed on the cell surface (Figure 4-D), indicating that some of the antibodies have been cleaved and secreted into the conditioned medium.

**Figure 5:**
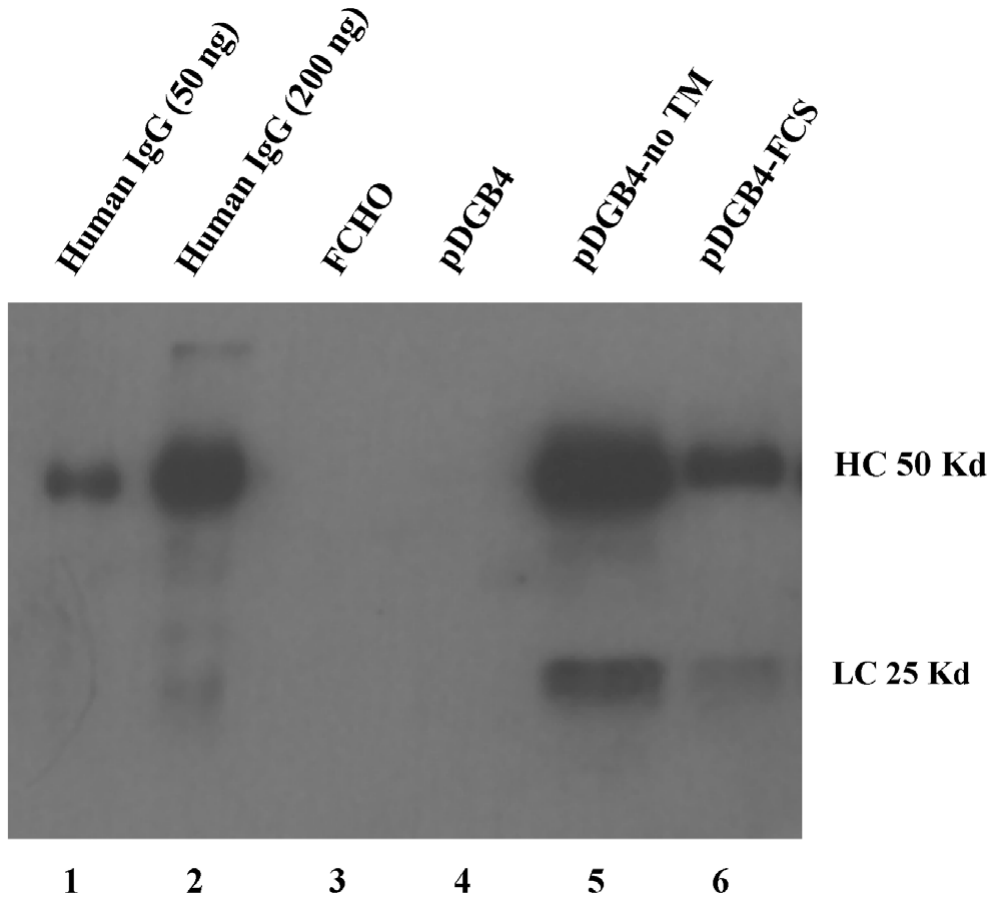
Western blot analysis of antibodies in conditioned media. Conditioned media were collected from stable transfected Flip-In CHO (FCHO) cells and concentrated 10-fold. Ten µl each were analyzed by electrophoresis with 10% SDS-PAGE gel. Lane 1: human IgG 50 ng; lane 2: human IgG 200 ng; lane 3: FCHO; lane 4: pDGB4; lane 5: pDGB4-noTM; and lane 6: pDGB4-FCS.

To confirm this inference, Western blotting was performed (Figure 5). The results show the absence of antibodies in the conditioned medium from FCHO (Lane 3) and pDGB4 (Lane 4), but presence of antibodies in the media from pDGB4-noTM (Lane 5) and pDGB4-FCS (Lane 6). Compared to the signal from pDGB4-noTM (Lane 5), the signal from pDGB4-FCS (Lane 6) was much weaker. To calculate the expression level of secreted antibodies from vector pDGB4-FCS and pDGB4-noTM, we analyzed the density of the heavy chain band in each lane. Based on the IgG standards in lanes 1 and 2 in Figure 5, the concentrations of lanes 4 and 5 were about 250 mg and 119 mg, respectively, and the calculated concentration in the conditioned media was 1.6 ng per µl for pDGB4-FCS and 3.3 ng per µl for pDGB4-noTM. These results fully agree with those from our FACS analysis and suggest that insertion of FCS has successfully and efficiently manipulated the dual cassette vector so that it can simultaneously express antibodies in both membrane-bound form for FACS analysis and secreted form for other experiments which use soluble antibodies.

## Discussion

Although past display technologies have been effectively used in a wide variety of applications, the quantity and quality of the libraries constructed have not yet met current needs for more efficient development of antibody drugs [22]. As the need for therapeutic antibodies for treatment of human diseases increases, platforms will transition more towards mammalian cell display, requiring corresponding advancement in all aspects of library construction. We reported the development of a unique mammalian display platform [13]-[18]. In our platform, a trans-membrane (TM) domain was fused to the 3′end of heavy chain in frame to display full-length antibody on mammalian cell surface. By site specific integration of the expression vector in the genome, each cell expresses only antibodies with single specificity. Because the light chain contains no anchor sequence and can only be detected when it has been correctly assembled with heavy chain on cell surface, the strength of the signal of light chain on cell surface will more accurately represent the expression level of full-length antibodies on cell surface. This platform shows advantages over past display techniques. In addition to reducing time and increasing efficiency in library construction, the display system also addresses the common problem of generating both membrane-bound antibodies for screening and soluble antibodies for function evaluation. After screening and selecting antibodies with high binding capabilities by mammalian display platform, the antibodies themselves, in general, cannot be used in functional assays since the antibody molecules are still attached to cell membranes. Therefore, to obtain antibodies in soluble form, the genes that encode the antibodies must be removed from the library vector and re-cloned into a new vector which contains no TM so that the antibodies expressed will be secreted into the cultured medium in a soluble form. This process requires multiple steps and multiple vectors. In contrast, our vector pDGB4-FCS requires only one-step cloning and can simultaneously express antibodies on the cell surface, as well as in the culture medium. The human antibodies on the cell surface can even detect very low concentrations of PE-conjugated anti-human kappa chain antibodies (one-seventh of the manufacturer's suggested dose; Figure 4). Our results have also demonstrated a sensitivity of detection at 1-2 µg per ml of human antibodies in conditioned medium (Figure 5). Therefore, the insertion of FCS into our vector allows for the selection of high-affinity antibodies through FACS and direct use of culture medium from the cells for functional assays. This use of furin for cleaving in combination with the transmembrane domain for anchoring for simultaneous expression of secreted and membrane-bound antibodies has not been reported before.

Future improvements to this method could be made by focusing on manipulating FCS. Since FCS is also inserted through enzyme digestion, we could remove it and try altering its RIRR amino acid sequence to determine if an increase or decrease occurs in the ratio of membrane-bound to soluble antibodies. These potential changes might provide solutions when more or less soluble forms of antibodies are needed for some experiments.

In conclusion, we have developed a vector for simultaneous expression of antibodies in both membrane-bound form and soluble form for functional screening and selecting of full- length monoclonal antibodies with high expression and affinity.
